# Sudden cardiac death while waiting: do we need the wearable cardioverter-defibrillator?

**DOI:** 10.1007/s00392-022-02003-4

**Published:** 2022-03-19

**Authors:** Carsten Israel, Ingo Staudacher, Christophe Leclercq, Giovanni Luca Botto, Daniel Scherr, Andreas Fach, Firat Duru, Maura M. Zylla, Hugo A. Katus, Dierk Thomas

**Affiliations:** 1 Department of Medicine, Division of Cardiology, Evangelisches Klinikum Bethel, Bielefeld, Germany; 2 Department of Cardiology, Medical University Hospital Heidelberg, Im Neuenheimer Feld 410, 69120 Heidelberg, Germany; 3 Heidelberg Center for Heart Rhythm Disorders, University Hospital Heidelberg, Heidelberg, Germany; 4 University of Rennes, CHU Rennes, LTSI-UMR1099, Rennes, France; 5 Ospedale di Circolo Rho, ASST Rhodense, Milan, Italy; 6 Division of Cardiology, Department of Internal Medicine, Medical University of Graz, Graz, Austria; 7 Klinikum Links der Weser, Department of Cardiology, Bremen, Germany; 8 Division of Cardiology, University Heart Center Zurich, Zurich, Switzerland; 9 Center for Integrative Human Physiology, University of Zurich, Zurich, Switzerland; 10 DZHK (German Center for Cardiovascular Research), Partner Site Heidelberg/Mannheim, University of Heidelberg, Heidelberg, Germany

**Keywords:** Wearable cardioverter-defibrillator, Sudden cardiac death, Ventricular tachycardia, Myocardial infarction, Implantable cardioverter-defibrillator

## Abstract

Sudden cardiac death (SCD) is the most frequent cause of cardiovascular death in industrialized nations. Patients with cardiomyopathy are at increased risk for SCD and may benefit from an implantable cardioverter-defibrillator (ICD). The risk of SCD is highest in the first months after myocardial infarction or first diagnosis of severe non-ischemic cardiomyopathy. On the other hand, left ventricular function may improve in a subset of patients to such an extent that an ICD might no longer be needed. To offer protection from a transient risk of SCD, the wearable cardioverter-defibrillator (WCD) is available. Results of the first randomized clinical trial investigating the role of the WCD after myocardial infarction were recently published. This review is intended to provide insight into data from the VEST trial, and to put these into perspective with studies and clinical experience. As a non-invasive, temporary therapy, the WCD may offer advantages over early ICD implantation. However, recent data demonstrate that patient compliance and education play a crucial role in this new concept of preventing SCD.

**Supplementary Information:**

The online version contains supplementary material available at 10.1007/s00392-022-02003-4.

## Prevention of sudden cardiac death: do we protect patients at risk?

Cardiovascular disease is the most common cause of death in industrialized nations. For instance, in 2016, cardiovascular disease caused 37% of all deaths in Germany (338,687 of 910,902), compared to 25% caused by any cancer [1]. Among patients with cardiovascular disease, sudden cardiac death (SCD) is the most frequent cause of death. SCD was noted on 13% of death certificates in the United States in 2016 (366,494 of 2,744,248), suggesting that 1 of 7.5 individuals in the United States die of SCD [2]. Even in the era of acute revascularization of myocardial infarction and treatment of heart failure with beta blockers, angiotensin receptor blockers, aldosterone antagonists, and neprilysin inhibitors, SCD is still the most frequent cause of death in patients with heart failure. In the PARADIGM-HF trial, 1251 deaths (81% of all deaths) were ascribed to cardiovascular causes. Of these, 45% were categorized as SCD compared to 27% of patients who died from progressive heart failure [3]. Of note, SCD was reduced in the group of patients treated with sacubitril/valsartan compared to the control group by 20%.

As shown in the German MONICA project in 1999, the outcome following out-of-hospital resuscitation for SCD is poor [4]. Return of spontaneous circulation was achieved in less than 50% of patients; of these, only 9% survived to hospital admission, and only 2% survived for more than 28 days. This has not significantly changed in recent reports on survival after out-of-hospital cardiac arrest [5].

Given the low survival rates of out-of-hospital cardiac arrest, primary prevention of SCD by the implantable cardioverter-defibrillator (ICD) has been positively evaluated in the MADIT [6, 7] and SCD-HeFT [8] trials. Therefore, symptomatic patients with a left ventricular ejection fraction (LVEF) of ≤ 35% currently have a class I (ischemic etiology) indication for an ICD. Owing to conflicting data about the need for primary prevention in non-ischemic heart failure patients, there is a class IIa indication for an ICD in these cases [9].

The risk of SCD in the first month after myocardial infarction is particularly high in patients with a low LVEF, and with an incidence of approximately 2–2.5% within only 30 days—10 times higher than annual mortality after the first year post-infarction [10]. Therefore, attempts have been made to protect patients as early as possible after myocardial infarction. The DINAMIT [11] and IRIS [12] trials randomized patients 6–40 and 5–31 days after a myocardial infarction associated with LVEF of 35% or lower to receive either an ICD or optimal medical treatment alone. Both trials showed a significant reduction of SCD by the ICD (-58% and -45%), albeit without any effect on all-cause mortality. The significant reduction in SCD by the ICD constituted an important observation, since it provided evidence that almost 50% of SCD cases post-MI were not arrhythmia related as suggested by data from the VALIANT trial [13]. These results have been explained by the “conversion theory”: patients with severe, progressive heart failure after myocardial infarction may develop ventricular tachyarrhythmias that can be terminated by the ICD. This phenomenon is thought to “shift” the mode of death from “sudden” to “non-sudden” in a significant number of patients. Similar effects have been seen in the MADIT II trial where patients (enrolled at least 1 month after myocardial infarction) with an ICD had lower all-cause mortality than patients without an ICD, however, at the cost of higher relative non-sudden cardiac mortality in ICD patients [14]. Therefore, guidelines demand a waiting period of at least 6 weeks after myocardial infarction before an ICD may be implanted.

This raises the question of whether it is safe for patients with a severely reduced left ventricular function to wait for therapy optimization before ICD implantation. Results in favour of early protection by a defibrillator can be derived from registry data. In the Cleveland Clinic registry, patients with a LVEF ≤ 35% who underwent percutaneous coronary intervention (PCI) or coronary artery bypass graft (CABG) surgery and had no ICD or wearable defibrillator showed a 90 day mortality of 8% (10% after PCI and 6% after CABG) [15]. The U. S. National Cardiovascular Data Registry reported a 90 day mortality of 32% for patients with PCI for ST elevation myocardial infarction with a LVEF ≤ 35% and age > 65 years [16]. Furthermore, the TRIUMPH registry reported that two-third of patients showing LVEF < 40% after myocardial infarction did not receive an echocardiographic reassessment within 6 months to help determine the eligibility for an ICD, mostly due to sub-optimal referral structures [17]. However, as early ICD implantation has not been shown to reduce overall mortality in randomized trials like IRIS und DINAMIT [11, 12] and has not been proven to provide benefit for patients at risk during the early phase after myocardial infarction, this period constitutes a therapeutic “gap” with no appropriate therapies at hand.

In patients with ischemic or non-ischemic cardiomyopathy, the ESC guidelines recommend a waiting time of at least 3 months after initiating optimal medical treatment [9]. In case of persistent LVEF ≤ 35% in combination with heart failure symptoms of at least NYHA functional class II, ICD implantation is recommended [9]. However, SCD risk and SCD prevention by the ICD were highest within these first 3 months of waiting time in the DEFINITE trial [18]. Patients with an ICD had a significant all-cause mortality benefit if they were randomized to ICD implantation within 3 months after diagnosis, whereas they did not benefit if non-ischemic cardiomyopathy was diagnosed (and treated) remotely. Similarly, the Heart Muscle Disease Registry of Triest [19] showed that the risk of SCD is highest in the first three months following the initial diagnosis of dilated cardiomyopathy.

In summary, available trials did not show a benefit of ICD therapy on total mortality in patients with severe left ventricular dysfunction early after myocardial infarction or after a first diagnosis of non-ischemic cardiomyopathy. Therefore, guidelines recommend waiting times of at least 40 days after myocardial infarction and at least 90 days on optimized treatment of non-ischemic cardiomyopathy. However, SCD risk during these waiting and drug titration periods is higher than during the later course of the patient and his heart disease, and patients are exposed to a considerable risk while waiting for reassessment. The growing number of effective drugs in heart failure render medical treatment more successful but also more complex and may require even more time on optimal medical treatment until a significant improvement in LVEF has to be excluded. This can be beneficial for patients who show a delayed improvement of heart failure obviating the need for an ICD but an individual disaster for patients who die from SCD during the waiting time. Automated external defibrillators for home use had no effect on total mortality [20]. To reduce the risk for SCD during these phases of stabilization and therapy optimization, avoiding the additional risks of an invasive procedure, a temporary therapeutic intervention such as a wearable cardioverter-defibrillator (WCD) may be useful. Based on this hypothesis, the VEST trial was initiated.

## The wearable cardioverter-defibrillator (WCD)

The wearable cardioverter-defibrillator (LifeVest^®^, Zoll Medical Corp., Pittsburgh, PA, US) has been available since 2001 as a non-invasive therapy for “temporary bridging” in patients with a potentially transient high risk of SCD. ECG and defibrillator electrodes are integrated within a vest garment consisting of straps. ECG electrodes continuously record and analyze a two-channel ECG. When a potentially life-threatening arrhythmia is detected, tactile, visual and acoustic alarms are initiated to warn the patient prior to the application of a shock. As long as the patient is conscious, therapy can be delayed by pressing a response button. If the patient has lost consciousness and does not delay WCD therapy, a defibrillation shock is delivered. During one episode of arrhythmia, a maximum of five shocks can be delivered. Via a transmitter within the charger unit, arrhythmia episodes are recorded by the device and automatically sent to a secured network (LifeVest^®^ Network) that can be accessed by the physician. Patients are advised to seek emergency medical support in case of a WCD therapy. To ensure an adequate patient reaction when the signal tone is emitted and to guarantee correct handling and functioning of the WCD, training and educating the patient are of particular importance. Furthermore, careful selection of patients who understand and follow the instructions is crucial as about 10–15% [21] of potential WCD patients are not capable of operating it correctly.

## Published experience: non-randomized studies

Since its introduction into clinical practice, more than 30,000 patients have been included into retrospective and prospective studies on the use of the WCD. An overview of registry data and studies is summarized in Supplementary Table 1. The main finding of prospective and retrospective registry data was high effectiveness of a WCD shock in case of ventricular arrhythmia. The average wear time as the most important measure of compliance was > 20 h/day. The rates of WCD therapies greatly varied between 1 and 12% (reaching up to 22% in cardiac sarcoidosis [22]) during the prescription period. A recent meta-analysis by Masri et al. [23] revealed appropriate shocks for ventricular tachyarrhythmias in 5% of patients over a period of only 3 months. There were no differences between patients with an indication for primary or secondary prevention of SCD. Compliance was consistently high with > 20 h per day, only 4 out of 28 studies showing slightly lower compliance rates.

## Randomized VEST trial

The VEST trial [24] was the first randomized, controlled, multicenter study on the WCD. It was an investigator-initiated trial, enrolling patients in the early post-myocardial infarction period with a reduced LVEF of ≤ 35%. Patients were included within 7 days of discharge from hospital. Patients allocated to the WCD group received the WCD for a follow-up period of 90 days as well as medication for heart failure according to guidelines. The control group received heart failure guideline-directed medication alone. ICD implantations, except for secondary prevention, and crossover were not permitted by the study design. The primary analysis plan was to perform an intention-to-treat analysis (ITT) and a secondary weighted sensitivity analysis excluding patients who could not be clearly classified. Initially, total mortality has been defined as the primary endpoint. Due to slow patient enrolment, the endpoint was later changed to ʻdeath by sudden cardiac death or VTʼ. Total mortality remained a secondary endpoint, in addition to non-sudden death, hospital admissions, compliance with wearing the device, and side effects. A total of 2302 patients were included in the study. Using 2:1 randomization, 1524 patients received the WCD and 778 were allocated to the control group. A subgroup of 2.8% of patients randomized to WCD refused this treatment after initial consent to participate in the trial. Unpermitted crossover from the control to the WCD group was recorded in 2.6% of patients who received a WCD in spite of study protocol regulations. Additionally, 5.7% of the control group received ICD implantation during the follow-up period (4.4% as protocol deviations). In the WCD group, 4.4% underwent ICD implantation (2.8% outside the protocol). The average WCD wear time in an intention-to-treat analysis was 14 h per day, counting all patients randomized to the WCD group whether they used it or not. The median wear time yielded 18 h/day. In this group, however, the WCD was worn on any given day only by 81% of the patients. In 20 patients (1.4%), VT/VF events were successfully terminated by the WCD. Of these, 14 patients were still alive after 90 days of follow-up. One patient, initially allocated to the control group, received a WCD against protocol and was treated by an appropriate WCD discharge due to an arrhythmia event. An unnecessary WCD shock was avoided by 4.5% of patients via use of the response button. Inappropriate shocks were delivered to 0.6% of patients.

**Fig. 1:**
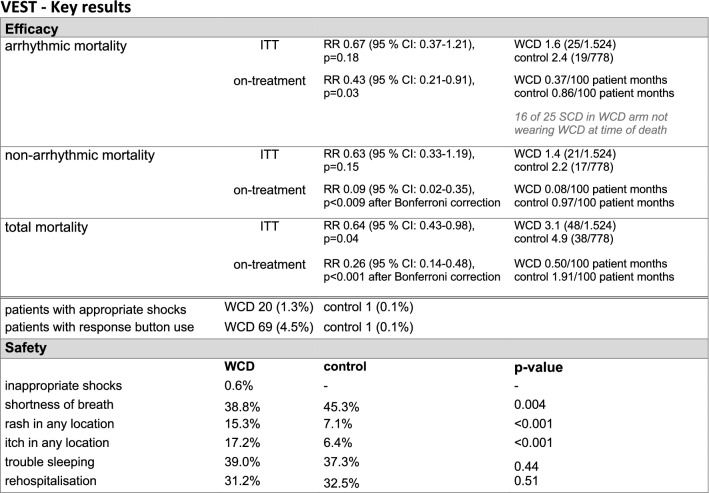
Key results from the VEST Trial. *ITT* intention-to-treat, *CI* confidence interval

Regarding the primary outcome of arrhythmic death, according to ITT analysis, there was no statistically significant difference between groups (*p* = 0.18). The primary endpoint occurred in 1.6% of patients in the WCD group and 2.4% of patients in the control group. The secondary endpoint of total mortality was significantly reduced in the WCD group, with a 36% relative risk reduction: 3.1% (WCD) versus 4.9% (control group) (*p* = 0.04), and an absolute reduction of 1.8%. Only 12 of the 48 patients (25%) who died in the group randomized to the WCD were actually wearing the WCD at the time of death, suggesting potential increase in efficacy with better compliance. The key results of the VEST trial are summarized in Fig. 1.

Of note, an initial, pre-specified as-treated analysis compared event rates per person-month between patients who were wearing the WCD and patients not wearing the WCD, independent of the randomization. This approach showed a significant relative risk reduction of the primary end point”arrhythmic death” by approximately 50% (*p* = 0.03). Both secondary endpoints “total mortality” (reduced by ~ 75%, *p *< 0.001) and “non-arrhythmic mortality” (reduced by almost 90%, *p *< 0.001) showed a relative risk advantage for wearing the WCD during the waiting period after myocardial infarction. Potential confounding effects were further assessed in subsequent as-treated and per-protocol analyses [25]. First, variability in wear time was corrected for in a per-protocol analysis that censored data inclusion at the last day of actual WCD wear time. This approach revealed significant reduction in total mortality and in arrhythmic death among WCD patients. Second, an as-treated sensitivity evaluation was employed to censor in-hospital time and events, with the intention to reduce the bias induced by lower WCD wear time and by higher mortality during hospitalization. No marked changes were observed with respect to overall mortality, arrhythmic death, and non-sudden death when all events were compared with out-of-hospital events only. Third, predictors of WCD wear time were identified and adjusted for in an additional as-treated study, revealing similar reduction of total mortality as well as arrhythmic and non-sudden death after adjustment. This post hoc multivariate analysis identified the following medical predictors associated with early WCD wear time discontinuation: LVEF ≤ 25% during the index myocardial infarction, prior diagnosis of diabetes or heart failure, and appropriate or inappropriate shock within 7 days prior to stopping of WCD wear [25]. As high wear time appears crucial for WCD effectiveness, these factors should be considered when selecting patients expected to benefit from WCD prescription post-myocardial infarction.

Results of the VEST trial require interpretation. Initially, the primary endpoint was all-cause mortality. However, since enrolment yielded only a little more than 200 patients in the first 2 years of the trial, the investigators felt that the aim of randomizing more than 4000 patients that were, according to assumptions, necessary to assess the effect of the WCD on all-cause mortality would not be achievable. Since the DINAMIT [11] and IRIS [12] trial showed no benefit of the ICD on all-cause mortality but a significant reduction of SCD mortality by approximately 50%, it was assumed that changing the primary endpoint to arrhythmic death would reduce the number of patients needed to just above 2000. However, the compliance in VEST was lower than expected and, therefore, the power of the study was substantially reduced. There was no intervention or remote monitoring (in contrast to observational studies or clinical routine WCD use) to ensure compliance in patients randomized to the WCD. As a consequence, a significant group of patients never wore the WCD at all during the study period, and the number of patients leaving the WCD in their wardrobe increased while it decreased in all registries and observational studies with a feedback to the patient whenever wear times < 20 h were detected. This might explain the difference between median and mean wearing times, and the striking difference in mortality between ITT and as-treated analyses as well as the observation that 75% of patients who died in the WCD group actually were not using the device at the time of death. These aspects constitute major limitations regarding the study execution and have to be considered in the interpretation of the results.

Perhaps, the most unexpected finding of the VEST trial was the observation that the secondary endpoint all-cause mortality was apparently reduced by the WCD. This finding has to be considered hypothesis-generating and not confirmatory as the primary end point was not significantly different. Even though it can only be speculated what the underlying mechanism for this observation might be, it is reasonable to assume that the WCD affected some patients’ compliance. The positive effect of communication interventions and feedback in patients with heart failure has been shown in multiple studies [26]. No such effect on non-cardiac death was observed in DINAMIT [11] or IRIS [12], on the contrary, as non-sudden cardiac death was increased in these studies in patients with an ICD compared to patients without an ICD. This stimulates considerations that the conversion theory (patients with severe heart failure who are saved from SCD will die from progressive heart failure) which may explain the results of DINAMIT and IRIS may not apply to a treatment that reduces the risk of SCD and heart failure progression at the same time. No specific intervention against progression of heart failure was instituted in DINAMIT and IRIS beyond optimal medical care. However, the WCD that has to be put on actively every day may act as a reminder to take care of heart failure and thus may have an impact not only on sudden but also non-sudden cardiac death.

Due to a significant number of crossovers in both groups negatively influencing the primary outcome, a final assessment of the role of the WCD regarding SCD protection remains challenging. Furthermore, the role of the WCD as a diagnostic device may have been underestimated in most trials and could not be assessed according to the VEST trial design. The WCD can also be used to record non-sustained ventricular arrhythmias, symptom-related arrhythmias, bradycardia events or supraventricular arrhythmias which may be of consequence for the patient’s therapy. This function may improve risk stratification and clinical outcomes and should therefore be assessed in future WCD trials. Considering the results of the VEST study, which showed an arrhythmic risk between 2.5% and 2.9% within 3 months, and the fact that 797 patients died before agreeing to participate in the study, the use of a WCD in within this patient cohort could be useful.

In summary, the VEST trial investigated a relevant patient population with a need for SCD protection and the potential of the WCD to serve their needs. Its results have to be discussed in light of the inherent limitations due to the change of the primary endpoint during the course of the study, crossovers and problems with statistical power. To benefit from the WCD, it is, however, necessary to ensure long wear times. The VEST trial may serve as a basis for future investigations regarding the use of the WCD in high-risk patient populations for the protection from SCD and its possible role in risk stratification by arrhythmia detection.

## Guideline recommendations

European guidelines have recommended use of the WCD in selected patients. Since 2015, the WCD has been included in all relevant guidelines of international professional societies for cardiology and electrophysiology as option to combat SCD, particularly when the risk is transient (Table 1) [9, 27–33].

**Table 1:** Overview of current guidelines/recommendations for wearable cardioverter-defibrillator use published by professional societies

Indications/recommendations	American Heart Association/American College of Cardiology [27]	European Society of Cardiology [9, 31]	German Cardiac Society [28]	Austrian Society of Cardiology [33]
After explantation, if reimplantation is not possible (e.g. infection)	IIa/B	IIb/C		IIa/C
Patients on the waiting list for a heart transplant without an ICD	IIb/B	IIb/C		IIa/C
Myocarditis and LVEF ≤ 35% and/or malignant arrhythmias	IIb/B	IIa/C	IIa	IIa/C
Patients with expected improvement of left ventricular function in non-ischaemic cardiomyopathy	IIb/B	IIb/C	IIb	IIb/C
Ischaemic cardiomyopathy and LVEF ≤ 35% (40 days after myocardial infarction, 40 days before and after PTCA, 90 days after surgical revascularization)	IIb/B	IIb/C	IIb	IIb/C
Potential prophylactic indication, definite diagnosis not yet established				IIb/C
Patients with HF who are at risk of sudden cardiac death for a limited period or as a bridge to an implanted device		IIb/B		

## The WCD as a tool to improve therapy compliance and health awareness

Additional benefits of the WCD apart from terminating SCD have been suggested. In a single center study, Zishiri et al. [15] compared patients after PCI and CABG from a WCD registry, many of them with prior myocardial infarction, with patients not wearing a WCD. A significant mortality difference was detected in propensity-matched analyses in favour of the WCD group that exceeded the mere effect of appropriate WCD shocks, suggesting increased therapy compliance or clinically relevant diagnostic information as hypothesized in the VEST trial. Considering that in a similar patient population, the two randomized controlled ICD trials DINAMIT and IRIS independently showed significantly higher non-arrhythmic mortality in the ICD group, these results warrant further exploration [11, 12].

## The WCD within health care systems

In the US, the WCD was licensed by the FDA in 2001. In the same year it received CE certification in Europe. In Germany, the WCD is listed in the therapeutic appliances list of technical aids of the National Association of Statutory Health Insurance Funds; in Switzerland the WCD is listed in the Swiss List of Medical Aids and Devices (MiGeL) of the Federal Office of Public Health; and in France, it is included in the List of Reimbursable Products and Services of the French Health Authority. An overview of the funding status in Europe is provided in Supplementary Table 2. Further countries with regulated funding of the WCD are Japan and Israel. In several other countries, funding is currently being negotiated*.*

The WCD is rented worldwide for a monthly fixed service rate that includes all costs and provided services. The VEST trial, that did not include any services once the WCD was handed over to the patient, suggests that provision of these services is mandatory to ensure full functionality of the WCD within the context of individual health systems and to ensure optimal compliance by the patient. There are two publications covering economic aspects of the WCD [34, 35]. Both calculate the Incremental Cost-Effectiveness Ratio (ICER) within the context of the US health care system, one of them for patients following ICD explantation, the other for patients post-myocardial infarction with LVEF ≤ 35%. ICERs calculated vary between $20.300/life-year and $44.100/life-year. In addition, systematic literature reviews and Health Technology Assessments (HTA) have been conducted. The HTA by Ettinger et al. [36] (initially compiled as “Rapid HTA” within the framework of the EUnetHTA Network [37]) was discussed in two letters [38, 39] with respect to restricted study inclusion criteria and the choice of comparators. Specifically, the potential limitation that a focus group analysis that was presented in the HTA included only a small group of five male patients following heart transplantation who had no experience or knowledge regarding the WCD was highlighted.

## Conclusion

The transient risk of SCD during the waiting period after myocardial infarction with a severely impaired left ventricular function is substantial. Current guidelines stress the usefulness of these waiting periods but do not clarify how SCD can be avoided during this time period. The DINAMIT and IRIS trials suggest that patients saved from SCD by an ICD had a higher non-sudden cardiac mortality, i.e. they may subsequently die from heart failure-related death, and therefore, all-cause mortality cannot be reduced by implanting an ICD early after myocardial infarction. While the primary end point of the VEST trial was not significantly different between groups in the ITT analysis, the study may trigger two novel hypotheses. First, wearing a WCD was associated with a numerically reduced all-cause mortality to a larger extent than reductions of SCD rates. Thus, it may be hypothesized that the WCD could serve as tool to increase patients’ awareness of a heart disease and improve compliance, e. g. with live-style modification and heart failure therapy. Second, potential reductions in non-arrhythmic mortality related to better compliance, combined with a significant reduction of arrhythmic mortality, could result in reduced total mortality. These hypotheses derived from the VEST trial merit validation in future, prospective studies.

## Supplementary Information

Below is the link to the electronic supplementary material.

Supplementary file1 (DOCX 62 KB)

## References

[1] Deutsches statistisches bundesamt (Federal Statistical Office Germany), https://www.destatis.de/DE/ZahlenFakten/GesellschaftStaat/Gesundheit/Todesursachen/Todesursachen.html, acceSsed February 24, 2019

[2] Centers for disease control and prevention, national center for health statistics. Multiple cause of death, 1999–2016. CDC WONDER Online Database [database online]. Released December 2017. Atlanta. http://wonder.cdc.gov/mcdicd10.html, Accessed September 11, 2018 9:57:28 PM

[3] Desai AS, McMurray JJ, Packer M, Swedberg K, Rouleau JL, Chen F, Gong J, Rizkala AR, Brahimi A, Claggett B, Finn PV, Hartley LH, Liu J, Lefkowitz M, Shi V, Zile MR, Solomon SD (2015). Effect of the angiotensin-receptor-neprilysin inhibitor LCZ696 compared with enalapril on mode of death in heart failure patients. Eur Heart J.

[4] Löwel H, Hörmann A, Gustomzyk J, Keil U (1999). Epidemiology of sudden cardiac death: what has changed? Results of the MONICA Augsburg myocardial infarction register 1985–95. Herzschr Elektrophys.

[5] Kragholm K, Wissenberg M, Mortensen RN, Hansen SM, Malta Hansen C, Thorsteinsson K, Rajan S, Lippert F, Folke F, Gislason G, Kober L, Fonager K, Jensen SE, Gerds TA, Torp-Pedersen C, Rasmussen BS (2017). Bystander efforts and 1-year outcomes in out-of-hospital cardiac arrest. N Engl J Med.

[6] Moss AJ, Hall WJ, Cannom DS, Daubert JP, Higgins SL, Klein H, Levine JH, Saksena S, Waldo AL, Wilber D, Brown MW, Heo M (1996). Improved survival with an implanted defibrillator in patients with coronary disease at high risk for ventricular arrhythmia. Multicenter automatic defibrillator implantation trial investigators. N Engl J Med.

[7] Moss AJ, Zareba W, Hall WJ, Klein H, Wilber DJ, Cannom DS, Daubert JP, Higgins SL, Brown MW, Andrews ML (2002). Multicenter automatic defibrillator implantation trial III. Prophylactic implantation of a defibrillator in patients with myocardial infarction and reduced ejection fraction. N Engl J Med.

[8] Bardy GH, Lee KL, Mark DB, Poole JE, Packer DL, Boineau R, Domanski M, Troutman C, Anderson J, Johnson G, McNulty SE, Clapp-Channing N, Davidson-Ray LD, Fraulo ES, Fishbein DP, Luceri RM, Ip JH (2005). Sudden cardiac death in heart failure trial I. Amiodarone or an implantable cardioverter-defibrillator for congestive heart failure. N Engl J Med.

[9] McDonagh TA, Metra M, Adamo M, Gardner RS, Baumbach A, Böhm M, Burri H, Butler J, Čelutkienė J, Chioncel O, Cleland JGF, Coats AJS, Crespo-Leiro MG, Farmakis D, Gilard M, Heymans S, Hoes AW, Jaarsma T, Jankowska EA, Lainscak M, Lam CSP, Lyon AR, McMurray JJV, Mebazaa A, Mindham R, Muneretto C, Francesco Piepoli M, Price S, Rosano GMC, Ruschitzka F, Kathrine Skibelund A; ESC Scientific Document Group (2021). 2021 ESC Guidelines for the diagnosis and treatment of acute and chronic heart failure. Eur Heart J.

[10] Solomon SD, Zelenkofske S, McMurray JJ, Finn PV, Velazquez E, Ertl G, Harsanyi A, Rouleau JL, Maggioni A, Kober L, White H, Van de Werf F, Pieper K, Califf RM, Pfeffer MA (2005). Valsartan in Acute myocardial infarction trial I. Sudden death in patients with myocardial infarction and left ventricular dysfunction, heart failure, or both. N Engl J Med.

[11] Hohnloser SH, Kuck KH, Dorian P, Roberts RS, Hampton JR, Hatala R, Fain E, Gent M, Connolly SJ, DINAMIT Investigators (2004). Prophylactic use of an implantable cardioverter-defibrillator after acute myocardial infarction. N Engl J Med.

[12] F, Lubinski A, Rosenqvist M, Habets A, Wegscheider K, Senges J, IRIS Steinbeck G, Andresen D, Seidl K, Brachmann J, Hoffmann E, Wojciechowski D, Kornacewicz-Jach Z, Sredniawa B, Lupkovics G, Hofgartner Investigators (2009). Defibrillator implantation early after myocardial infarction. N Engl J Med.

[13] Pouleur AC, Barkoudah E, Uno H, Skali H, Finn PV, Zelenkofske SL, Belenkov YN, Mareev V, Velazquez EJ, Rouleau JL, Maggioni AP, Køber L, Califf RM, McMurray JJ, Pfeffer MA, Solomon SD; VALIANT Investigators (2010). Pathogenesis of sudden unexpected death in a clinical trial of patients with myocardial infarction and left ventricular dysfunction, heart failure, or both. Circulation.

[14] Goldenberg I, Moss AJ, Hall WJ, McNitt S, Zareba W, Andrews ML, Cannom DS; Multicenter Automatic Defibrillator Implantation Trial (MADIT) II Investigators (2006). Causes and consequences of heart failure after prophylactic implantation of a defibrillator in the multicenter automatic defibrillator implantation trial II. Circulation.

[15] Zishiri ET, Williams S, Cronin EM, Blackstone EH, Ellis SG, Roselli EE, Smedira NG, Gillinov AM, Glad JA, Tchou PJ, Szymkiewicz SJ, Chung MK (2013). Early risk of mortality after coronary artery revascularization in patients with left ventricular dysfunction and potential role of the wearable cardioverter defibrillator. Circ Arrhythm Electrophysiol.

[16] Weintraub WS, Grau-Sepulveda MV, Weiss JM, Delong ER, Peterson ED, O’Brien SM, Kolm P, Klein LW, Shaw RE, McKay C, Ritzenthaler LL, Popma JJ, Messenger JC, Shahian DM, Grover FL, Mayer JE, Garratt KN, Moussa ID, Edwards FH, Dangas GD (2012). Prediction of long-term mortality after percutaneous coronary intervention in older adults: results from the national cardiovascular data registry. Circulation.

[17] Miller AL, Gosch K, Daugherty SL, Rathore S, Peterson PN, Peterson ED, Ho PM, Chan PS, Lanfear DE, Spertus JA, Wang TY (2013). Failure to reassess ejection fraction after acute myocardial infarction in potential implantable cardioverter/defibrillator candidates: insights from the translational research investigating underlying disparities in acute myocardial infarction patients’ health status (TRIUMPH) registry. Am Heart J.

[18] Kadish A, Schaechter A, Subacius H, Thattassery E, Sanders W, Anderson KP, Dyer A, Goldberger J, Levine J (2006). Patients with recently diagnosed nonischemic cardiomyopathy benefit from implantable cardioverter-defibrillators. J Am Coll Cardiol.

[19] Massa L, Della Barca F, Vitali-Serdoz L, Perkan A, Moretti M, Sabadini G, Di Lenarda A, Sinagra G (2003). Heart failure etiology and long-term response to medical treatment. The heart muscle disease registry of triest. Eur J Heart Fail.

[20] Bardy GH, Lee KL, Mark DB, Poole JE, Toff WD, Tonkin AM, Smith W, Dorian P, Packer DL, White RD, Longstreth WT Jr, Anderson J, Johnson G, Bischoff E, Yallop JJ, McNulty S, Ray LD, Clapp-Channing NE, Rosenberg Y, Schron EB; HAT Investigators (2008). Home use of automated external defibrillators for sudden cardiac arrest. N Engl J Med.

[21] Barraud J, Cautela J, Orabona M, Pinto J, Missenard O, Laine M, Thuny F, Paganelli F, Bonello L, Peyrol M (2017). Wearable cardioverter defibrillator: bridge or alternative to implantation?. World J Cardiol.

[22] Skowasch D, Ringquist S, Nickenig G, Andrie R (2018). Management of sudden cardiac death in cardiac sarcoidosis using the wearable cardioverter defibrillator. PLoS ONE.

[23] Masri A, Altibi AM, Erqou S, Zmaili MA, Saleh A, Al-Adham R, Ayoub K, Baghal M, Alkukhun L, Barakat AF, Jain S, Saba S, Adelstein E (2019). Wearable cardioverter-defibrillator therapy for the prevention of sudden cardiac death: a systematic review and meta-analysis. JACC Clin Electrophysiol.

[24] Olgin JE, Pletcher MJ, Vittinghoff E, Wranicz J, Malik R, Morin DP, Zweibel S, Buxton AE, Elayi CS, Chung EH, Rashba E, Borggrefe M, Hue TF, Maguire C, Lin F, Simon JA, Hulley S, Lee BK, Vest Investigators (2018). Wearable cardioverter-defibrillator after myocardial infarction. N Engl J Med.

[25] Olgin JE, Lee BK, Vittinghoff E, Morin DP, Zweibel S, Rashba E, Chung EH, Borggrefe M, Hulley S, Lin F, Hue TF, Pletcher MJ (2020). Impact of wearable cardioverter-defibrillator compliance on outcomes in the VEST trial: As-treated and per-protocol analyses. J Cardiovasc Electrophysiol.

[26] Ruppar TM, Cooper PS, Mehr DR, Delgado JM, Dunbar-Jacob JM (2016). Medication adherence interventions improve heart failure mortality and readmission rates: systematic review and meta-analysis of controlled trials. J Am Heart Assoc.

[27] Al-Khatib SM, Stevenson WG, Ackerman MJ, Bryant WJ, Callans DJ, Curtis AB, Deal BJ, Dickfeld T, Field ME, Fonarow GC, Gillis AM, Granger CB, Hammill SC, Hlatky MA, Joglar JA, Kay GN, Matlock DD, Myerburg RJ, Page RL (2018). 2017 AHA/ACC/HRS Guideline for management of patients with ventricular arrhythmias and the prevention of sudden cardiac death: executive summary. Circulation.

[28] Deneke T, Borggrefe M, Hindricks G, Kirchhof P, Kuck KH, Stellbrink C, Eckardt L (2018). Kommentar zu den ESC-Leitlinien 2015 „Ventrikuläre Arrhythmien und Prävention des plötzlichen Herztodes“. Kardiologe.

[29] Reek S, Burri H, Roberts PR, Perings C, Epstein AE, Klein HU; EHRA Scientific Documents Committee (as external reviewers): Lip G, Gorenek B, Sticherling C, Fauchier L, Goette A, Jung W, Vos MA, Brignole M, Elsner C, Dan GA, Marin F, Boriani G, Lane D, Blomström-Lundqvist C, Savelieva I (2017). The wearable cardioverter-defibrillator: current technology and evolving indications. Europace.

[30] Piccini JP Sr, Allen LA, Kudenchuk PJ, Page RL, Patel MR, Turakhia MP; American Heart Association Electrocardiography and Arrhythmias Committee of the Council on Clinical Cardiology and Council on Cardiovascular and Stroke Nursing (2016). Wearable cardioverter-defibrillator therapy for the prevention of sudden cardiac death: a science advisory from the American heart association. Circulation.

[31] Priori SG, Blomström-Lundqvist C, Mazzanti A, Blom N, Borggrefe M, Camm J, Elliott PM, Fitzsimons D, Hatala R, Hindricks G, Kirchhof P, Kjeldsen K, Kuck KH, Hernandez-Madrid A, Nikolaou N, Norekvål TM, Spaulding C, Van Veldhuisen DJ; ESC Scientific Document Group (2015). 2015 ESC guidelines for the management of patients with ventricular arrhythmias and the prevention of sudden cardiac death: the task force for the management of patients with ventricular arrhythmias and the prevention of sudden cardiac death of the European society of cardiology (ESC). Endorsed by: association for European paediatric and congenital cardiology (AEPC). Eur Heart J.

[32] Gronda E, Bourge RC, Costanzo MR, Deng M, Mancini D, Martinelli L, Torre-Amione G, O'Hara ML, Chambers S (2006). Heart rhythm considerations in heart transplant candidates and considerations for ventricular assist devices: international society for heart and lung transplantation guidelines for the care of cardiac transplant candidates–2006. J Heart Lung Transplant.

[33] Scherr D, Mörtl D, Keller H, Ebner C (2017). Positionspapier zum einsatz des tragbaren kardioverter defibrillators. J Kardiologie.

[34] Healy CA, Carrillo RG (2015). Wearable cardioverter-defibrillator for prevention of sudden cardiac death after infected implantable cardioverter-defibrillator removal: a cost-effectiveness evaluation. Heart Rhythm.

[35] Sanders GD, Owens DK, Hlatky MA (2015). Potential cost-effectiveness of wearable cardioverter-defibrillator early post myocardial infarction. J Innov Card Rhythm Manag.

[36] Ettinger S, Stanak M, Szymanski P, Wild C, Hacek R, Ercevic D, Grenkovic R, Huic M (2017). Wearable cardioverter defibrillators for the prevention of sudden cardiac arrest: a health technology assessment and patient focus group study. Med Devices (Auckl).

[37] Ettinger S, Stanak M, Huic M, Hacek R, Ercevic D, Grenkovic R (2016) Wearable cardioverter-defibrillator (WCD) therapy in primary and secondary prevention of sudden cardiac arrest in patients at risk. EUnetHTA Rapid HTA; version 1.4

[38] Sperzel J, Staudacher I, Goeing O, Stockburger M, Meyer T, Goncalves ASO, Sydow H, Schoenfelder T, Amelung VE (2018). Critical appraisal concerning “Wearable cardioverter defibrillators for the prevention of sudden cardiac arrest: a health technology assessment and patient focus group study”. Med Devices (Auckl).

[39] Sperzel J, Staudacher I, Goeing O, Stockburger M, Meyer T, Goncalves ASO, Sydow H, Schoenfelder T, Amelung V (2018). Comments on the authors' reply to the critical appraisal concerning “Wearable cardioverter defibrillators for the prevention of sudden cardiac arrest: a health technology assessment and patient focus group study”. Med Devices (Auckl).

