# Supplementary material for: Sudden cardiac death while waiting: do we need the wearable cardioverter-defibrillator?
Source: Clin Res Cardiol. 2022 Mar 19;111(11):1189–97. doi: 10.1007/s00392-022-02003-4 (PMC9622539; doi:10.1007/s00392-022-02003-4)
Supplement: Supplementary file 1 — Supplementary file1 (DOCX 62 KB) [file 392_2022_2003_MOESM1_ESM.docx]

**Supplementary Material to:**

**Sudden cardiac death while waiting: Do we need the wearable cardioverter-defibrillator?**

Carsten Israel^1^, Ingo Staudacher^2,3^, Christophe Leclercq^4^, Giovanni Luca Botto^5^, Daniel Scherr^6^, Andreas Fach^7^, Firat Duru^8,9^, Maura M. Zylla^2,3,10^, Hugo A. Katus^2,3,10^, Dierk Thomas^2,3,10^

^1^Evangelisches Klinikum Bethel, Department of Medicine, Division of Cardiology, Bielefeld, Germany; ^2^Department of Cardiology, Medical University Hospital Heidelberg, Germany; ^3^Heidelberg Center for Heart Rhythm Disorders, University Hospital Heidelberg, Germany; ^4^University of Rennes, CHU Rennes, LTSI-UMR1099, Rennes, France ; ^5^Ospedale di Circolo Rho, ASST Rhodense, Milan, Italy ; ^6^Division of Cardiology, Department of Internal Medicine, Medical University of Graz, Austria; ^7^Klinikum Links der Weser, Department of Cardiology, Bremen, Germany; ^8^Division of Cardiology, University Heart Center Zurich, Switzerland; ^9^Center for Integrative Human Physiology, University of Zurich, Switzerland; ^10^DZHK (German Center for Cardiovascular Research), partner site Heidelberg/Mannheim, University of Heidelberg, Germany

**Corresponding author:** Dierk Thomas, MD, FAHA, FEHRA, FESC, FHRS; Department of Cardiology, University of Heidelberg, Im Neuenheimer Feld 410, 69120 Heidelberg, Germany; Tel.: ++49 6221 568855; Fax: ++49 6221 565514; E-Mail: dierk.thomas@med.uni-heidelberg.de

**Supplementary Table 1.** Overview of published registry data and studies

| **Study design** | **Reference** | **Year** | **Patients (n)** | **Indication** | **Appropr. shocks** | **Inappr. shocks** | **Compliance (hours/day)** | **Total wear time** | **WCD followed by ICD impl.** | **a) First shock success rate b) Survival rate after wearing WCD** | **a) Death while wearing WCD b) Death during wearing period** |
| --- | --- | --- | --- | --- | --- | --- | --- | --- | --- | --- | --- |
| **Retro-spective study** | Barraud (1) | 2017 | 24 | post-MI w. PCI | 1 (1 pat., 4.2%) | 0 | mean 21.5, median 23.5 | n.r. | n.d., 50% need no ICD after Follow Up | a)+b) 100% | a)+b) 0 |
|  | Beiert (2) | 2017 | 114 | div. | 19 (11 pat., 9.6%) | 0 | median 23.1 | median 52.0d | 57 (51.4%) | n.r. | a) 1 |
|  | Castro (3) | 2017 | 21 | lead extraction | 1 (4.8%) | 0 | mean 22.0 | mean 51.6+ 16.9d | 100% | 1 (100%) | a)+b) 0 |
|  | Chung (4) | 2010 | 3569 | div. | 80 (in 59 pat.) (2.2%) | 67 (1.9%) | median 19.9 + 4.7 | 52.6 + 69.9 d | n.r. | a) 99% | a) 20 |
|  | Collins (5) | 2010 | 184 (81 < 18 years of age, 103 19-21 y.o.a.) | children compared with young adults | 0/5 | 1/1 | n.r. | n.r. | n.r. | n.r. | n.r. |
|  | Duncker (6) | 2017 | 49 | PPCM | 6 (12%) | 0 | mean 21.4+3.3 | mean 120+106d | n.r. | n.r. | n.r. |
|  | Duncker/ PROLONG (7) | 2017 | 156 | div. | 12 (11 pat.) (7%) | 0 | mean 21.7+4.0 | mean 101+89d | 42 ICD/ 17 CRT-D | n.r. | 5 (all at prolonged wearing period) |
|  | Ellen-bogen (8) | 2017 | 8056 | lead extraction | 348 (334 pat./4.1%) | 159 (2%) | n.r. | median 50d | 81% | b) 30d post event survival 81%/ 93% overall/ 94% in those with WCD shock | b) 9% |
|  | Epstein (9) | 2013 | 8453 | Post-MI | 146 (133 pat./1.6%) | 114 (106/1.2%) | median 21.8 | average 69+61d / median 57d) | n.r. | a) 86% b) 91% (1.4%) | n.r. |
|  | Heimeshoff (10) | 2018 | 100 | following cardiac surgery (59 pat. CABG) | 3 (3%) | 0 | median 23.5 | median 60d | 25 (25%) | b) 100% | a)+b) 0 |
|  | Klein (11) | 2010 | 354 | div. | 27 (7.6%) | 0 | 21.3 (72% worn 22-24h) | median 106d | 43% | n.r. |  |
|  | Opreanu (12) | 2015 | 122 | candidates for heart  trans-plantation | 7 | 2 | average 17+7 (median 20) | median 39d | 42% (51) | b) 91% | b) 11 |
|  | Owen (13) | 2018 | 10 | long-QT-syndrome | 1 | 0 | n.r. | median 24d | n.r. | n.r. | n.r. |
|  | Salehi (14) | 2016 | 127 | CM based on excess alcohol use and other CM | 9 (5.5%) | 18 | median 18.0 | median 51d | 23.6% (reason for discontinuing WCD) | a) 100% | a)+b) 0 |
|  | Saltzberg (15) | 2012 | 266 (107/159) | PPCM/ NICM | 0/2 (1 pat.) | 0/0 | n.r. | mean 124/96 | n.r. | 100% | 0/11 |
|  | Sasaki (16) | 2017 | 50 | div. | 6 (12%) (3 pat./7.5%) | n.r. | median 23.7 | median 16d | 27 (54%) | a) 5 (83.3%) | n.r. |
|  | Singh (17) | 2015 | 525 | NICM/ICM | 6 (1.1%) | 5 (1%) | median 22 | median 61d | 125 (46%) ICM/107 (42%) NICM | n.r. | n.r. |
|  | Skowasch (18) | 2018 | 46 | cardiac sarcoidosis | 11 (10 pat., 21.7%) | n.r. | median 23.6 | median 33d | 23 (50%) | a) 100% | a)-b) 0 |
|  | Spar (19) | 2018 | 455 | pediatric pat. (median 15 (3-17) years old) | 6 (1.3%) | 2 (0.4%) | median 20.6 | median 33d | n.r. | a) 100%  b) 98.5% | a) 0  b) 7 (1.5%) |
|  | Tanawutti-wat (20) | 2014 | 97 | ICD explantation | 4 | 2 | median 20 | median 21d |  | b) 91.2% |  |
|  | Wäßnig (21) | 2016 | 6043 | div. | 120 (2%) | 26 (0.4%) | median 23.1 | median 59d | n.r. | a) 88 (94%), b) 87 (93%) | a)+b)8 |
|  | Zishiri (22) | 2013 | 4958 (809 vs. 4149) | post MI: PCI and CABG vs. control groups | 18/1.3% (11 pat.) | 13(0.6%) | n.r. | n.r. | 32%/30%(PCI/CABG) | a) 67%, b) 98%/97% (PCI/CABG) (90d) | n.r. |
|  | Zylla (23) | 2018 | 106 | div. | 3 (2.8%) | 2 (1.9%) | mean 22.7 | median 58.5d | 29.6% | n.r. | a)+b) 1 (0.9%) |
|  | Barsheshet (24) | 2017 | 75 | ICM/NICM | 1 (1.3%) | 0 | median 18 | median 59d | n.r. | n.r. | n.r. |
|  | Kovacs (25) | 2020 | 456 | ICM (45.2%), NICM (25.2%), bridge to ICD/TX (8.1%), congenital heart disease (3.3%), unspecified (18.2%) | 17 (3.7%) | 0 | median 22.6 | n.r. | 46.5% | n.r. | n.r. |
| **Pro-spective study** | Veltmann (26) | 2020 | 781 | HF (NICM/ICM) | 13 (1.3%) | 2 (0.3%) | mean 20.3 | mean 75d | 37% | 100%/- | 2 (died from terminal HF, one from non-cardiac cause) |
|  | Garcia (27) | 2020 | 1157 | explant (10.3%), bridge to TX (7.6%), ICM after early post-MI/ ICM after recent PCI (82.1%) | 18 (1.6%), 4.7% arrhythmic events detected | 0.7% | median 23.4 (86% wore the WCD >20h/d, 25% wore the WCD >23.75h/d) | Median 62d | 50.6% | a)100%, b)100% | b) 2.1% |
|  | Rosen-kaimer (28) | 2020 | 153 | div. | 6 (4%) | 1 (0.7%) | 21.3 | 65.1d | 42% | n.d. | 2 (septic shock, pulseless electr. activ. and low output after hemodialysis) |
|  | Daimee (29) | 2018 | 1732 | div.// age: >65/<65 | 6.92%/2.37% | n.r. | median 22.8/22.3h | s. WEARIT II | 41.8/36.5 | n.r. | n.r. |
|  | Duncker (30) | 2014 | (12)9 | PPCM | 4 (3 pat.) | no inappropriate shock was mentioned | mean 22.0+2.4 | median 81d, mean 133+103d | 1 | a) 100% | a)+b) 0 |
|  | Erath (31) | 2017 | 102 | div. | 4 (3.9%) | 2 (2%) | median 23 | median 54d | 56 (55%) | n.r. | n.r. |
|  | Erath (32) | 2018 | 130 | TMP (20) vs. div. (110) | 0 | 0 | n.r. | n.r. | 3/20 vs. 40/110 | 0 | a)+b) 0 |
|  | Feldmann WEARIT/ BIROAD (33) | 2004 | 289 | div. | 6 + 2 unsuccessful (total 2.8%) | 6 (2%) | mean 19.2/21 | median 3.1 months | n.r. | n.r. | n.r. |
|  | Kao/WIF (34) | 2012 | 89 | HF | 0 | 0 | average 19.5+4.6, median 21.8(7-277) | average 75+58d | 34.1% | b) 100% | a)+b) 0 |
|  | Kondo (35) | 2015 | 24 | Post-Mi | 3 (2 pat., 8.3%) | 0 | median 23.1 | median 33d | 14 | a) 100% | a)+b) 0 |
|  | Kutyifa/ WEARIT II (36) | 2015 | 2000 | div. | 30 (22 pat., 1.1%) | 10 (0.5%) | median 22.5 | median 90d | 840(42%) | a) 100% | a)+b) 3 (0.2%) |
|  | Kutyifa/ WEARIT II (37) | 2018 | 2000 | div.// WCD use <90d vs. >90d | 19 (1.9%) vs. 3 (0.3%) | n.r. | 22.2h vs. 22.5h | 981 (49%) >90d/1019 <90d | 33% vs. 47% | a)100% | n.d., see WEARIT II |
|  | Odeneg (38) | 2017 | 720 | div | 44 (25 pat., 3.5%) | 4 (3 pat., 0.4%) | median 23.5 | median 55d | 51% | n.r. | n.r. |
|  | Rao (39) | 2011 | 162 (43/119) | congenital structural heart disease and inherited arrhythmias | 3/2% (0%/2%) | 7/3% (0%/3%) | median 19 (both groups) | 27/29 d | n.r. | n.r. | b) 7% |
|  | Reek (40) | 2003 | 12 | EP testing, history of MI | 22 | 0 | n.a. | n.a. | n.a. | a) 100% b) 100% | none |
|  | Reek (41) | 2002 | 12 | div. | 22 (induced VA) | 0 | n.r. | n.r. | n.r. | n.r. | n.r. |
|  | Röger (42) | 2018 | 105 | div. | 5 (4.8%) | 1 (1.0%) | median 21.5+3.5 | median 68.8+ 50.4d | 51.4% | n.r. | n.r. |
| **Meta-analysis** | Nguyen (43) | 2018 | Meta-analysis 19.882 | div. | 1.7% | 0.9% | 17-24, 7 studies report >20h | > 3 months in 9 studies | n.r. | 95.5%  (successful termination) | total mortality 0.2 VT/VF related; all-cause mortality 1.4% |
|  | Masri (44) | 2019 | 33242 | meta-analysis/div. | 2.65% ICM/2.32%NICM | 0.66% | n.d. | n.d. | n.d. | 95.50% | a) 0.232 % |
| **RCT** | Olgin (45, 46) | 2018 | 2302 | post MI | 1.3% (+ 0.1% in the control group) | 0.6% | ITT: median 18.0, mean 14.0;  if WCD was worn: median 23.9 (day 1)-23.2 (day 90), mean 22.3+4.3 (day 1)-20.3+5.7 (day 90) | mean 48.3d | n.d. | n.d. | b) total mort.: 3.1% WCD group/4.9% Ctrl.; SCD: 1.6% WCD group, 2.4% Ctrl. |

CABG, coronary artery bypass graft; CM, cardiomyopathy; div., diverse; HF, heart failure; ICD, implantable cardioverter defibrillation; ICM, ischemic cardiomyopathy; MI, myocardial infarction; n.r., no data reported; n.d., not defined; NICM, non-ischemic cardiomyopathy; pat, patients; PCI, percutaneous coronary intervention; PPCM, peripartum cardiomyopathy; RCT, randomized controlled trial; TX, heart transplantation; VF, ventricular fibrillation; VT, ventricular tachycardia; WCD, wearable cardioverter-defibrillator

**SupplementaryTable 2:** Overview of current funding regulation within Europe

| **Country** | **Funding** | **Register** |
| --- | --- | --- |
| Germany | Regulated | HMV |
| France | Regulated | LPPR |
| Luxembourg | Regulated | Fichier B7 |
| Switzerland | Regulated | MiGeL |
| Italy | Unregulated | - |
| Austria | Unregulated | - |
| Poland | In process | - |
| Sweden | Unregulated | - |
| Great Britain | Unregulated | - |

HMV, „Hilfsmittelregister“; LPPR, „liste des produits et prestations remboursés“; MiGEL, „Mittel und Gegenständeliste“

**Supplementary References**

1. Barraud J, Cautela J, Orabona M, Pinto J, Missenard O, Laine M, Thuny F, Paganelli F, Bonello L, Peyrol M. Wearable cardioverter defibrillator: Bridge or alternative to implantation? World J Cardiol 2017;9:531-8
2. Beiert T, Malotki R, Kraemer N, Stockigt F, Linhart M, Nickenig G, Schrickel JW, Andrie RP. A real world wearable cardioverter defibrillator experience - Very high appropriate shock rate in ischemic cardiomyopathy patients at a European single-center. J Electrocardiol 2017;50:603-9
3. Castro L, Pecha S, Linder M, Vogler J, Gosau N, Meyer C, Willems S, Reichenspurner H, Hakmi S. The wearable cardioverter defibrillator as a bridge to reimplantation in patients with ICD or CRT-D-related infections. J Cardiothorac Surg 2017;12:99
4. Chung MK, Szymkiewicz SJ, Shao M, Zishiri E, Niebauer MJ, Lindsay BD, Tchou PJ. Aggregate national experience with the wearable cardioverter-defibrillator: event rates, compliance, and survival. J Am Coll Cardiol 2010;56:194-203
5. Collins KK, Silva JN, Rhee EK, Schaffer MS. Use of a wearable automated defibrillator in children compared to young adults. Pacing Clin Electrophysiol 2010;33:1119-24
6. Duncker D, Westenfeld R, Konrad T, Pfeffer T, Correia de Freitas CA, Pfister R, Thomas D, Furnkranz A, Andrie RP, Napp A, Schmitt J, Karolyi L, Wakili R, Hilfiker-Kleiner D, Bauersachs J, Veltmann C. Risk for life-threatening arrhythmia in newly diagnosed peripartum cardiomyopathy with low ejection fraction: a German multi-centre analysis. Clin Res Cardiol 2017;106:582-9
7. Duncker D, Konig T, Hohmann S, Bauersachs J, Veltmann C. Avoiding untimely implantable cardioverter/defibrillator implantation by intensified heart failure therapy optimization supported by the wearable cardioverter/defibrillator-the PROLONG study. J Am Heart Assoc 2017;6:doi: 10.1161/JAHA.116.004512
8. Ellenbogen KA, Koneru JN, Sharma PS, Deshpande S, Wan C, Szymkiewicz SJ. Benefit of the wearable cardioverter-defibrillator in protecting patients after implantable-cardioverter defibrillator explant: results from the national registry. JACC Clin Electrophysiol 2017;3:243-50
9. Epstein AE, Abraham WT, Bianco NR, Kern KB, Mirro M, Rao SV, Rhee EK, Solomon SD, Szymkiewicz SJ. Wearable cardioverter-defibrillator use in patients perceived to be at high risk early post-myocardial infarction. J Am Coll Cardiol 2013;62:2000-7
10. Heimeshoff J, Merz C, Ricklefs M, Kirchhoff F, Haverich A, Bara C, Kuhn C. Wearable cardioverter-defibrillators following cardiac surgery-a single-center experience. Thorac Cardiovasc Surg 2019;67:92-7
11. Klein HU, Meltendorf U, Reek S, Smid J, Kuss S, Cygankiewicz I, Jons C, Szymkiewicz S, Buhtz F, Wollbrueck A, Zareba W, Moss AJ. Bridging a temporary high risk of sudden arrhythmic death. Experience with the wearable cardioverter defibrillator (WCD). Pacing Clin Electrophysiol 2010;33:353-67
12. Opreanu M, Wan C, Singh V, Salehi N, Ahmad J, Szymkiewicz SJ, Thakur RK. Wearable cardioverter-defibrillator as a bridge to cardiac transplantation: A national database analysis. J Heart Lung Transplant 2015;34:1305-9
13. Owen HJ, Bos JM, Ackerman MJ. Wearable cardioverter defibrillators for patients with long QT syndrome. Int J Cardiol 2018;268:132-6
14. Salehi N, Nasiri M, Bianco NR, Opreanu M, Singh V, Satija V, Jhand AS, Karapetyan L, Safadi AR, Surapaneni P, Thakur RK. The wearable cardioverter defibrillator in nonischemic cardiomyopathy: a US national database analysis. Can J Cardiol 2016;32:1247
15. Saltzberg MT, Szymkiewicz S, Bianco NR. Characteristics and outcomes of peripartum versus nonperipartum cardiomyopathy in women using a wearable cardiac defibrillator. J Card Fail 2012;18:21-7
16. Sasaki S, Shoji Y, Ishida Y, Kinjo T, Tsushima Y, Seno M, Nishizaki F, Itoh T, Izumiyama K, Yokota T, Yokoyama H, Yamada M, Horiuchi D, Kimura M, Higuma T, Tomita H, Okumura K. Potential roles of the wearable cardioverter-defibrillator in acute phase care of patients at high risk of sudden cardiac death: A single-center Japanese experience. J Cardiol 2017;69:359-63
17. Singh M, Wang NC, Jain S, Voigt AH, Saba S, Adelstein EC. Utility of the wearable cardioverter-defibrillator in patients with newly diagnosed cardiomyopathy: a decade-long single-center experience. J Am Coll Cardiol 2015;66:2607-13
18. Skowasch D, Ringquist S, Nickenig G, Andrie R. Management of sudden cardiac death in cardiac sarcoidosis using the wearable cardioverter defibrillator. PLoS One 2018;13:e0194496
19. Spar DS, Bianco NR, Knilans TK, Czosek RJ, Anderson JB. The US experience of the wearable cardioverter-defibrillator in pediatric patients. Circ Arrhythm Electrophysiol 2018;11:e006163
20. Tanawuttiwat T, Garisto JD, Salow A, Glad JM, Szymkiewicz S, Saltzman HE, Kutalek SP, Carrillo RG. Protection from outpatient sudden cardiac death following ICD removal using a wearable cardioverter defibrillator. Pacing Clin Electrophysiol 2014;37:562-8
21. Wassnig NK, Gunther M, Quick S, Pfluecke C, Rottstadt F, Szymkiewicz SJ, Ringquist S, Strasser RH, Speiser U. Experience with the wearable cardioverter-defibrillator in patients at high risk for sudden cardiac death. Circulation 2016;134:635-43
22. Zishiri ET, Williams S, Cronin EM, Blackstone EH, Ellis SG, Roselli EE, Smedira NG, Gillinov AM, Glad JA, Tchou PJ, Szymkiewicz SJ, Chung MK. Early risk of mortality after coronary artery revascularization in patients with left ventricular dysfunction and potential role of the wearable cardioverter defibrillator. Circ Arrhythm Electrophysiol 2013;6:117-28
23. Zylla MM, Hillmann HAK, Proctor T, Kieser M, Scholz E, Zitron E, Katus HA, Thomas D. Use of the wearable cardioverter-defibrillator (WCD) and WCD-based remote rhythm monitoring in a real-life patient cohort. Heart Vessels 2018;33:1390-402
24. Barsheshet A, Kutyifa V, Vamvouris T, Moss AJ, Biton Y, Chen L, Storozynsky E, Wan C, Szymkiewicz SJ, Goldenberg I. Study of the wearable cardioverter defibrillator in advanced heart-failure patients (SWIFT). J Cardiovasc Electrophysiol 2017;28:778-84
25. Kovacs B, Reek S, Sticherling C, Schaer B, Linka A, Ammann P, Brenner R, Krasniqi N, Müller A, Dzemali O, Kobza R, Grebmer C, Haegelt L, Berg J, Mayer K, Schläpfer J, Domenichini G, Reichlin T, Roten L, Burri H, Eriksson U, Saguner A, Steffel J, Duru F. Use of the the wearable cardioverter-defibrillator-the Swiss experience. Swiss Med Wkly 2020;150:w20343
26. Veltmann C, Winter S, Duncker D, Jungbauer C, Wäßnig N, Geller C, Erath J, Goeing O, Perings C, Ulbrich M, Roser M, Husser D, Gansera L, Soezener, Malur F, Block M, Fetsch M, Kutyifa V, Klein H. Protected risk stratification with the wearable cardioverter-defibrillator: results from the WEARIT-II-EUROPE registry. Clin Res Cardiol 2021;110:102-13
27. Garcia R, Combes N, Defaye P, Narayanan K, Guedon-Moreau L, Boveda S, Blangy H, Bouet J, Briand F, Chevalier P, Cottin Y, Da Costa A, Degand B, Deharo J, Eschalier R, Exramiana F, Goralski M, Guy-Moyat B, Guyomar Y, Herminda J, Jourda F, Lellouche N, Mahfoud M, Manenti V, Mansourati J, Martin A, Pasquié J, Ritter P, Rollin A, Tibi T, Yalioua A, Gras D, Sadoul N, Poit O, Leclercq C, Marijon E. Wearable cardioverter-defibrillator in patients with a transient risk of sudden cardiac death: the WEARIT-France cohort study. Europace 2021;23:73-81
28. Rosenkaimer S, El-Battrawy I, Dreher T, Gerhards S, Röger S, Kuschyk J, Borggrefe M, Akin I. The wearable cardioverter-defibrillator: experience in 153 patients and a long-term follow-up. J Clin Med 2020;9:893
29. Daimee UA, Vermilye K, Moss AJ, Goldenberg I, Klein HU, McNitt S, Zareba W, Kutyifa V. Experience with the wearable cardioverter-defibrillator in older patients: results from the Prospective Registry of Patients Using the Wearable Cardioverter-Defibrillator. Heart Rhythm 2018;15:1379-86
30. Duncker D, Haghikia A, Konig T, Hohmann S, Gutleben KJ, Westenfeld R, Oswald H, Klein H, Bauersachs J, Hilfiker-Kleiner D, Veltmann C. Risk for ventricular fibrillation in peripartum cardiomyopathy with severely reduced left ventricular function-value of the wearable cardioverter/defibrillator. Eur J Heart Fail 2014;16:1331-6
31. Erath JW, Vamos M, Sirat AS, Hohnloser SH. The wearable cardioverter-defibrillator in a real-world clinical setting: experience in 102 consecutive patients. Clin Res Cardiol 2017;106:300-6
32. Erath JW, Vamos M, Benz AP, Hohnloser SH. Usefulness of the WCD in patients with suspected tachymyopathy. Clin Res Cardiol 2018;107:70-5
33. Feldman AM, Klein H, Tchou P, Murali S, Hall WJ, Mancini D, Boehmer J, Harvey M, Heilman MS, Szymkiewicz SJ, Moss AJ, WEARIT investigators and coordinators; BIROAD investigators and coordinators. Use of a wearable defibrillator in terminating tachyarrhythmias in patients at high risk for sudden death: results of the WEARIT/BIROAD. Pacing Clin Electrophysiol 2004;27:4-9
34. Kao AC, Krause SW, Handa R, Karia D, Reyes G, Bianco NR, Szymkiewicz SJ. Wearable defibrillator use in heart failure (WIF): results of a prospective registry. BMC Cardiovasc Disord 2012;12:123
35. Kondo Y, Linhart M, Andrie RP, Schwab JO. Usefulness of the wearable cardioverter defibrillator in patients in the early post-myocardial infarction phase with high risk of sudden cardiac death: A single-center European experience. J Arrhythm 2015;31:293-5
36. Kutyifa V, Moss AJ, Klein H, Biton Y, McNitt S, MacKecknie B, Zareba W, Goldenberg I. Use of the wearable cardioverter defibrillator in high-risk cardiac patients: data from the Prospective Registry of Patients Using the Wearable Cardioverter Defibrillator (WEARIT-II Registry). Circulation 2015;132:1613-9
37. Kutyifa V, Vermilye K, Daimee UA, McNitt S, Klein H, Moss AJ. Extended use of the wearable cardioverter-defibrillator in patients at risk for sudden cardiac death. Europace 2018;20(FI2):f225-32
38. Odeneg T, Ebner C, Mortl D, Keller H, Dirninger A, Stix G, Foger B, Grimm G, Steinwender C, Gebetsberger F, Stuhlinger M, Mastnak B, Haider C, Manninger M, Scherr D. Indications for and outcome in patients with the wearable cardioverter-defibrillator in a nurse-based training programme: results of the Austrian WCD Registry. Eur J Cardiovasc Nurs 2019;18:75-83
39. Rao M, Goldenberg I, Moss AJ, Klein H, Huang DT, Bianco NR, Szymkiewicz SJ, Zareba W, Brenyo A, Buber J, Barsheshet A. Wearable defibrillator in congenital structural heart disease and inherited arrhythmias. Am J Cardiol 2011;108:1632-8
40. Reek S, Meltendorf U, Geller JC, Wollbruck A, Grund S, Klein HU. The Wearable Cardioverter Defibrillator (WCD) for the prevention of sudden cardiac death -- a single center experience. Z Kardiol 2002;91:1044-52
41. Reek S, Geller JC, Meltendorf U, Wollbrueck A, Szymkiewicz SJ, Klein HU. Clinical efficacy of a wearable defibrillator in acutely terminating episodes of ventricular fibrillation using biphasic shocks. Pacing Clin Electrophysiol 2003;26:2016-22
42. Roger S, Rosenkaimer SL, Hohneck A, Lang S, El-Battrawy I, Rudic B, Tulumen E, Stach K, Kuschyk J, Akin I, Borggrefe M. Therapy optimization in patients with heart failure: the role of the wearable cardioverter-defibrillator in a real-world setting. BMC Cardiovasc Disord 2018;18:52
43. Nguyen E, Weeda ER, Kohn CG, D’Souza BA, Russo AM, Noreika S, Coleman CI. Wearable cardioverter-defibrillators for the prevention of sudden cardiac death: a meta-analysis. The Journal of Innovations in Cardiac Rhythm Management 2018;9:3151-62
44. Masri A, Altibi A, Erqou S, Zmaili M, Saleh A, Al-Adham R, Ayoub K, Baghal M, Alkukhun L, Barakat A, Jain S, Saba S, Adelstein E. Wearable cardiverter-defibrillator therapy for the prevention of sudden cardiac death: a systematic review and meta-analysis. JACC Clin Electrophysiol 2019;5:152-61
45. Olgin JE, Pletcher MJ, Vittinghoff E, Wranicz J, Malik R, Morin DP, Zweibek S, Buxton A, Elayi C, Chung E, Rashba E, Borggrefe M, Hue T, Maguire C, Lin F, Simon J, Hulley S, Lee B. Wearable cardioverter-defibrillator after myocardial infarction. New Engl J Med 2018;379:1205-15
46. Olgin JE, Lee B, Vittinghoff E, Morin DP, Zweibel S, Rashba E, Chung E, Borggrefe M, Hulley S, Lin F, Hue T, Pletcher MJ. Impact of wearable cardioverter‐defibrillator compliance on outcomes in the VEST trial: as‐treated and per‐protocol analyses. J Cardiovasc Electrophysiol 2020;31:1009-18
